# Combined training improves the diagnostic measures of sarcopenia and decreases the inflammation in HIV‐infected individuals

**DOI:** 10.1002/jcsm.12926

**Published:** 2022-02-09

**Authors:** Morteza Ghayomzadeh, Daniel Hackett, SeyedAhmad SeyedAlinaghi, Mohammad Gholami, Negin Hosseini Rouzbahani, Fabrício Azevedo Voltarelli

**Affiliations:** ^1^ Department of Exercise Science Murdoch University Perth Western Australia Australia; ^2^ Murdoch Applied Sports Science Laboratory Murdoch University Perth Western Australia Australia; ^3^ Physical Activity, Lifestyle, Ageing and Wellbeing Faculty Research Group, School of Health Sciences, Faculty of Medicine and Health The University of Sydney Sydney New South Wales Australia; ^4^ Iranian Research Center for HIV/AIDS, Iranian Institute for Reduction of High‐Risk Behaviors Tehran University of Medical Sciences Tehran Iran; ^5^ Department of Medical Microbiology, Faculty of Medicine AJA University of Medical Sciences Tehran Iran; ^6^ Department of Medical Immunology, Faculty of Medicine AJA University of Medical Sciences Tehran Iran; ^7^ Graduate Program in Health Sciences, Faculty of Medicine Federal University of Mato Grosso Cuiab'a Brazil

**Keywords:** Combined training, HIV, Wasting syndrome, Antiretroviral therapy, HIV‐related sarcopenia

## Abstract

**Background:**

HIV‐related sarcopenia is an emerging health issue that often remains undiagnosed and can lead to reduced quality of life, independence, and premature death if untreated. This study investigated the effects of a 6 month combined training (resistance plus aerobic exercise) (CT) intervention on diagnostic measures of sarcopenia, including grip strength, appendicular lean mass index (ALMI), and gait speed.

**Methods:**

Forty participants were randomized into either a CT group (*n* = 20; age = 38.3 ± 4.9 years) or a control group (CON; *n* = 20; age = 37.9 ± 5.1 years). Participants in the CT group performed three supervised sessions per week for 6 months, consisting of weekly reverse linear periodized resistance training followed by 20 min aerobic training. Participants in the CON group were instructed to continue with their current lifestyle habits. Assessments were completed at baseline and after 6 months. Statistical analyses were performed using a two‐way analysis of covariance (ANCOVA) adjusted for sex and preintervention values. Primary outcomes included grip strength, ALMI, and gait speed. Secondary outcomes were changes in levels of pro‐inflammatory cytokines (IL‐6 and TNF‐α), IGF‐1, and myostatin. Associations were explored between changes in inflammatory markers (IL‐6 and TNF‐α), gait speed, and ALMI with grip strength.

**Results:**

A significant increase in ALMI was found for CT compared with CON (0.29 ± 0.13 kg/m^2^ vs. −0.11 ± 0.14 kg/m^2^, respectively; *P* < 0.001). Significant improvements in grip strength (7.86 ± 8.50 kg for CT vs. −1.58 ± 2.47 kg for CON) and gait speed (0.16 ± 0.07 m/s^2^ for CT vs. −0.06 ± 0.52 m/s^2^ for CON; both *P* < 0.001) were also observed in CT compared with CON. Reduction in inflammatory biomarkers was found in CT compared with CON (IL‐6; TNF‐α, both *P* < 0.001). An increase in IGF‐1 (74.36 ± 56.64 pg/mm^3^ for CT vs. 7.19 ± 99 pg/mm^3^ for CON; *P* < 0.001) and a decrease in myostatin (−158.90 ± 62.03 pg/mm^3^ for CT vs. −43.33 ± 146.60 pg/mm^3^ for CON; *P* < 0.001) was found following CT compared with the CON group. Change in grip strength was correlated with changes in IL‐6 (*r* = −0.65, *P* < 0.001), TNF‐α (*r* = −0.63, *P* < 0.001), gait speed (*r* = 0.63, *P* < 0.001), and ALMI (*r* = 0.54, *P* = 0.001), but not IGF‐1 and myostatin. No adverse events were recorded, and compliance with the CT exercise sessions was high (>85%).

**Conclusions:**

Combined training appears to be an effective means to counteract sarcopenia and improve various inflammatory markers and growth hormones in people living with HIV.

## Introduction

People living with HIV (PLWH) develop age‐related co‐morbidities more frequently and at earlier ages than noninfected individuals. Of particular concern in this cohort is sarcopenia, characterized by loss of muscle function and mass.^1^, ^2^ There is a high prevalence of HIV‐associated sarcopenia in PLWH, and this condition is distinct from HIV wasting syndrome, which was common co‐morbidity in PLWH in the pre antiretroviral therapy (ART) era.^3^ Findings from a recent systematic review by Olivera *et al*.^1^ estimated the prevalence of sarcopenia in PLWH as 24.1% in those aged 35 to 60 years. These results were based on cross‐sectional studies that evaluated the prevalence of sarcopenia using measures that included appendicular muscle mass, muscle strength, and physical function.^1^ This proportion of PLWH with sarcopenia is greater than other clinical populations at risk, such as older adults with type 2 diabetes and chronic kidney diseases. Olivera *et al*.^1^ also suggested that lower income, BMI, and being male were associated with the higher incidence of sarcopenia.

Sarcopenia is strongly linked with adverse clinical outcomes, including increased hospitalization, falls, and premature death in different clinical cohorts, including PLWH.^2^ Furthermore, self‐reliance and the ability to perform activities of daily living are negatively affected in PLWH with sarcopenia,^2^ consequently increasing their risk of institutionalization. The aetiology of sarcopenia in PLWH is likely multifactorial and related to a combination of HIV‐specific complications (e.g. persistent chronic immune activation and inflammation) and lifestyle factors (e.g. smoking, drug and alcohol consumption, and malnutrition).^1^, ^4^ Of the limited strategies available to counteract sarcopenia, physical training is considered one of the best treatment options due to the low rate of side effects and low cost.^1^, ^4^ In particular, resistance training during the early stages of sarcopenia has been shown to yield notable improvement in the diagnostic measures of sarcopenia among older adults.^5^ Additionally, numerous clinical trials have shown the beneficial effects of different types of exercise training on the measures of sarcopenia in different clinical populations.^6^

Although extensive research has investigated the effects of exercise training on muscle mass,^7^, ^8^ strength,^9^, ^10^ and physical function^11^ in PLWH, there is limited research that has examined all three measures of sarcopenia in a single study. Furthermore, the current exercise‐based intervention studies in PLWH have ignored sex differences in response to the exercise training, particularly regarding changes in muscle mass and strength. Because the aetiology of sarcopenia in PLWH is likely different compared with sarcopenia in other clinical cohorts, the extrapolation of results from other populations may be inappropriate. Therefore, based on the paucity of research on this topic to date, exploring the effectiveness of different types of physical training on the diagnostic measures of sarcopenia (with consideration to sex) in PLWH is important.

The main aim of this study was to examine the effects of a 6 month combined training (resistance plus aerobic exercise) intervention on diagnostic measures of sarcopenia, including grip strength, appendicular lean mass index (ALMI), and gait speed in HIV‐infected men and women. Secondary outcomes included inflammatory markers and growth factors related to the development of sarcopenia. We hypothesized that combined training would favourably improve (i) measures of sarcopenia and (ii) the secondary outcomes presented above.

## Materials and methods

### Participants and study design

Forty HIV‐infected patients (20 men and 20 women) aged between 23 and 45 years [38 ± 7 years; mean ± standard deviation (SD)] were recruited from the Iranian Institute for Reduction of High‐Risk Behaviours and gave their written informed consent to participate in this study. This study was approved by the Tehran University of Medical Sciences (protocol no: IRCT20100601004076N21, TUMS) and registered in the Iranian Register of Clinical Trials (IRCT). The exercise for people living with HIV (EPLH) study was a 6 month, assessor‐blinded, randomized controlled trial and was conducted between October 2017 and December 2019. The primary study objective was to examine the effect of CT on bone loss in PLWH.^12^ This prespecified secondary analysis investigated the effect of CT on diagnostic measures of sarcopenia, including ALMI, grip strength, and gait speed. Additionally, exploring the correlation between changes in ALMI, inflammatory, and growth factors over the course of the study period.

### Sample size

A sample size of 40 participants was determined to provide sufficient power to detect meaningful effects based on primary and secondary outcomes of the main study, including the diagnostic measures of the sarcopenia separately. This was calculated (a priori) using G*Power software (v. 3.1.7) with considering type 1 error 5% and type 2 error of 20% (power = 80%). As this was a secondary analysis, the sample size was not modifiable, and we did not perform an a priori or post hoc sample size calculation. However, as mentioned earlier, because the diagnostic measures of sarcopenia were among the considered variables when calculating the sample size of the main study, the current study was deemed to have been adequately powered.

### Inclusion and allocation

Eligibility criteria included being aged 18–45 years, no active opportunistic infection or other acute clinical conditions within the past 3 months, no drug or alcohol addictions, a stable antiretroviral therapy (ART) regimen for >6 months, CD4 + count ≥300 cells per cubic millimetre, and undetectable viral load; and women needed to be in a premenopausal state. Participants were excluded if they were participating in regular physical activity during the previous 6 months, had a detectable viral load (>50 copies/mm^3^), had existing muscle or joint problems preventing the performance of an exercise, and taking any drug or therapy that can affect muscle mass such as anabolic steroids and dietary supplements.

**Figure 1:**
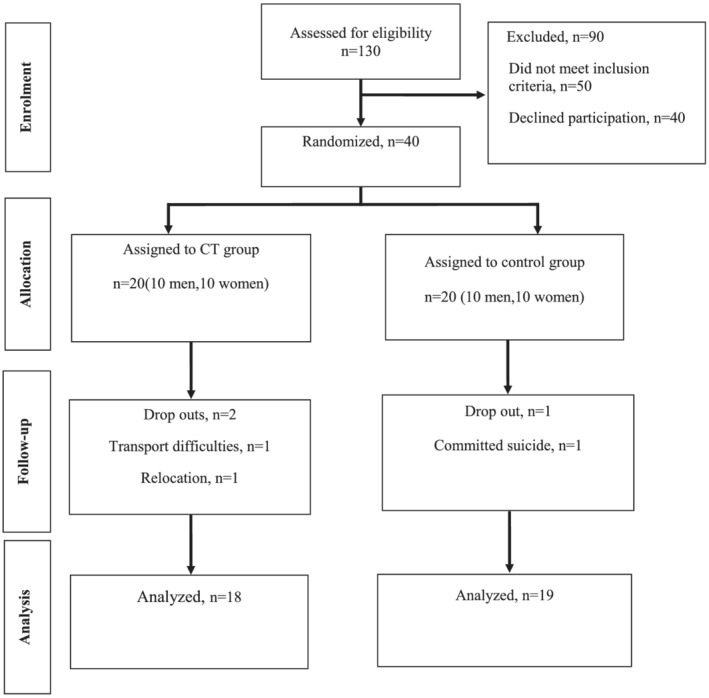
CONSORT diagram of the study shows the disposition of patients randomized to CT or control group for 6 months.

Eligible participants were randomly assigned to either CT (*n* = 20, 10 men, 10 women; 38.3 ± 4.9 years) or control group (CON; *n* = 20, 10 men, 10 women; 37.9 ± 5.1 years) in a 1:1 allocation ratio. Two participants in the CT and one in the CON did not complete the follow‐up assessments leaving 18 participants in CT and 19 in CON to complete the study (*Figure* 1). Participants randomized into CT attended supervised exercise sessions 3 days/week for 6 months. Participants in the CON were encouraged to continue their usual care without changing their daily habits, including their diet. Participants of both groups were asked to avoid taking dietary supplements during the study period. Verbal and written informed consent was obtained from participants before starting the study.

### Training protocol

The training protocol has been previously described in detail.^12^ Briefly, as shown in *Table* 1, participants who were randomized into the CT group were asked to perform three sets of eight resistance exercises (bench press, shoulder press, squat, leg extension, triceps extension, lat pull down, plank, and back extension) on 3 days (non‐consecutive) per week for 6 months. The training load was adjusted so that participants could perform sets of 8, 12, and 20 repetitions per exercise for each subsequent training day (i.e. weekly reverse linear periodized resistance). Training loads were increased when participants exceeded the prescribed number of repetitions. After the resistance training regimen, participants completed 20 min aerobic exercise on a motorized treadmill, consisting of a 5 min warm‐up followed by 15 min exercise at 65–80% of the age‐predicted target heart rate (THR). Heart rate was monitored during all training sessions (Polar© V800 Finland).

**Table 1:** Characteristics of the non‐linear resistance training protocol

Exercise	Day 1	Day 2	Day 3
Bench press	3 × 4–6	3 × 8–12	3 × 15–12
Squat	3 × 4–6	3 × 8–12	3 × 15–12
Shoulder press	3 × 4–6	3 × 8–12	3 × 15–12
Leg extension	3 × 4–6	3 × 8–12	3 × 15–12
Triceps extension	3 × 4–6	3 × 8–12	3 × 15–12
Lat pulldown	3 × 4–6	3 × 8–12	3 × 15–12
Plank	Until failure	Until failure	Until failure
Back extension	3 × 20	3 × 20	3 × 20
Rest between sets	2–3 min	1–2 min	1 min
Intensity	(80–85) % 1 RM	(60–80) % 1 RM	(50–65) % 1 RM

Data are presented as the number of sets and the targeted range of repetitions during training sessions.

### Familiarization period

Prior to commencing the training sessions, participants assigned to the CT group underwent a 2 week (six sessions) familiarization period facilitated by a trainer. Familiarization with resistance training involved three sets of 8–12 repetitions for all the prescribed exercises using light weights (i.e. 11–13 on the rating of perceived exertion—RPE scale).^13^ Following the familiarization period, participants performed one‐repetition maximum (1RM) testing according to the American College of Sports Medicine (ACSM) guidelines to determine the initial training loads.^14^

### Body composition

Body composition was assessed at baseline and follow‐up (6 months), after an overnight fast by a dual‐energy X‐ray absorptiometry (DEXA) machine (Hologic QDR 4500A scanner; Hologic Inc., Waltham, MA, USA) using a whole‐body scan. In a prone position, while clothes were removed down to the underwear and wearing a hospital gown, participants were scanned from head to toe, providing appendicular lean mass values of the upper and lower body. All scans were performed and analysed by the same technician. The intra‐rater reliability of the technician conducting the DEXA scans at the Imam Khomeini hospital complex nuclear medicine Laboratory (Tehran, Iran) was excellent, with a coefficient of 1%. The ALMI was calculated by dividing the total lean mass of the arms and legs (kg) by height (m^2^), according to the EWGSOP2 standard.^15^

### Blood sampling and biochemical analyses

A certified phlebotomist took whole blood samples (5 mL) from participants at the baseline and follow‐up assessments after an overnight fast and at least 48 h following the last training session. Whole blood was centrifuged (1500 rpm, 15 min, 4°C), and the resultant serum was stored (−80°C) in multiple 1.5 mL plastic tubes (Eppendorf©, Hamburg, Germany). Biochemical assessments of cytokine profile (IL‐6 and TNF‐α), IGF1, and myostatin were determined by enzyme‐linked immunosorbent assay (ELISA) kits as determined by the manufacturer (R&D System; Minneapolis, MN, USA). The biochemical assessments were conducted by a blinded technician in a licensed clinical laboratory within the Imam Khomeini Hospital.

### Physical performance

#### Grip strength

The grip strength of the dominant hand was measured by a dynamometer (Jamar ® hydraulic dynamometer) which could be adjusted to allow participants to grip the device with their middle two fingers formed a 90 degree angle.^16^ During the assessment, participants were instructed to keep their hands neutral and bend their elbow to 90 degrees between the upper arm and forearm. Participants were instructed to squeeze the dynamometer as hard as they could and hold it for 2 s. Three trials were completed with 1 min rest between trials, and the best performance was used for data analysis.

#### Gait speed

Participants' gait speed was measured by the timed up and go (TUG) test. Participants were required to stand up from a seated position on a chair, walk for 3 m, turn around, and sit down again. The time scored in the TUG test was converted to an estimate of gait speed by using the following formula: [6/(TUG time) × 1.62]. This equation has been validated and previously used by other researchers.^17^, ^18^

#### Diagnosis of sarcopenia

Participants were screened for sarcopenia using the most current criteria from the European Working Group on Sarcopenia in Older People (EWGSOP2).^15^ The presence of sarcopenia was evaluated by ALMI, grip strength, and gait speed. Based on the severity of the symptoms, this criterion categorized sarcopenia into three stages: pre‐sarcopenic, sarcopenic, and severe sarcopenic. The pre‐sarcopenic status was confirmed when grip strength fell below 27 kg for men and less than 16 kg for women. Sarcopenia status was confirmed if the pre‐sarcopenic condition was accompanied by low ALMI defined as below 7.0 and 5.5 kg/m^2^ for men and women, respectively. Severe sarcopenia was confirmed with low grip strength and ALMI as well as a gait speed below 0.8 m/s.

#### Adherence to the exercise programme

Adherence was calculated by the number of sessions the participant performed divided by the total number of sessions offered, multiplied by 100.

### Statistical analysis

The Shapiro–Wilk normality test was used to confirm the normality and homogeneity of variances.

A two‐way analysis of covariance (ANCOVA) adjusted for sex and preintervention values was performed to compare the change from baseline scores (Delta score, Δ). Due to the possible effects of sex on the measured variables and different cut of values for diagnosing sarcopenia in men and women, data for women and men were also analysed separately via the ANOVA. Within‐group analysis was performed using paired sample *t*‐tests. A partial correlation analysis (controlling for sex) was performed to examine whether changes in grip strength was associated with changes in ALMI, inflammatory markers, and gait speed. The strength of correlations was qualitatively assessed as small (*r* < 0.3), moderate (*r* > 0.3 to 0.5), strong (*r* > 0.5 to 0.7), very strong (*r* > 0.7 to 0.9), nearly perfect (*r* > 0.9), and perfect (*r* = 1.0). Statistical analyses were performed using SPSS® version 24 (IBM North America, New York, NY, USA). Throughout the manuscript, data are presented as mean (SD) or mean change (95% CI). An alpha level of significance was set at *P* < 0.05, except for the correlation analysis, which was set at *P* < 0.01 to help protect against potential type I errors.

## Results

The participant's characteristics, both stratified by sex and total, are presented in *Table* 2. There were no differences between CT and CON at baseline for age, BMI, ALMI, and gait speed. Compared with women, men were heavier (*P* < 0.004), taller (*P* < 0.003), and their ALMI was greater than females (*P* < 0.001) at the baseline. Additionally, baseline handgrip strength values in the dominant hand were greater for men compared with women (*P* < 0.001).

**Table 2:** Sample characterization at baseline

Characteristic	CON‐men (*n* = 10)	CT‐men (*n* = 10)	CON‐women (*n* = 10)	CT‐women (*n* = 10)	CON (*n* = 20)	CT (*n* = 20)	*P* value (between‐groups)
Age (years)	38.3 ± 5.6	36.2 ± 6.7	37 ± 7.3	36.3 ± 5.8	38.59 ± 8.94	36.3 ± 2.1	0.42
Years of infection	8.58 ± 2.3	8.63 ± 3.5	8.65 ± 6.5	9.23 ± 4.7	8.16 ± 2.24	8.2 ± 3.8	0.54
Years on ART	7.36 ± 1.2	7.48 ± 2.4	7.29 ± 2.3	8.67 ± 2.2	7.51 ± 2.3	8.61 ± 2.1	0.84
Current smoker, *n* (%)	5 (50)	6 (60)	3 (30)	2 (20)	8 (40)	8 (40)	—
Marital status, *n* (%)							
Single/widowed	4 (40)	5 (50)	4 (40)	4 (40)	8 (40)	9 (45)	—
Married/cohabitating	3 (30)	3 (30)	3 (30)	5 (50)	6 (30)	8 (40)	—
Divorced/separated	3 (30)	2 (20)	3 (30)	1 (10)	6 (30)	3 (15)	—
Anthropometry							
Height (cm)	174 ± 7.5	176 ± 5.3	168 ± 9.6	165 ± 5.6	170 ± 7.27	170 ± 7.3	0.001^a^
BMI (m/kg^2^)	25.23 ± 2.99	26.45 ± 3.33	24.69 ± 5.38	26.82 ± 2.64	25.07 ± 4.5	25.91 ± 3.12	0.30
HAART medications							
NRTI/NNRTI	10	10	10	10	20	20	—
INI	9	8	9	9	18	17	—
PI	1	2	1	1	2	3	—
Blood markers							
IL‐6 (pg/mm^3^)	8.52 ± 1.21	8.92 ± 0.76	9.17 ± 1.79	7.75 ± 0.66	8.79 ± 1.46	8.53 ± 0.91	0.56
TNF‐α (pg/mm^3^)	13.19 ± 2.42	13.54 ± 2.00	11.43 ± 2.64	14.25 ± 2.40	12.46 ± 2.59	13.78 ± 2.08	0.54
IGF‐1 (pg/mm^3^)	162.16 ± 101.26	150.92 ± 52.07	137.31 ± 73.79	146.49 ± 69.08	151.9 ± 89.2	122.78 ± 58.37	0.85
Myostatine (pg/mm^3^)	1165.12 ± 236	1428.04 ± 316	1282.31 ± 226	1275.68 ± 444	1213.4 ± 232	1377.2 ± 355.8	0.87
Measures of sarcopenia							
Handgrip strength (kg)	42.50 ± 7.98	44.50 ± 4.92	24.86 ± 5.96	26.80 ± 4.72	34.94 ± 5.91	35.67 ± 4.05	0.20^a^
Gait speed (m/s)	1.30 ± 0.07	1.27 ± 0.07	1.29 ± 0.06	1.31 ± 0.07	1.29 ± 0.07	1.28 ± 0.08	0.08
ALMI (kg/m^2^)	6.86 ± 0.46	6.59 ± 0.64	5.69 ± 0.53	5.87 ± 0.32	6.28 ± 0.55	6.25 ± 0.67	0.06^a^
Sarcopenia, *n* (%)^β^							
Pre‐sarcopenic	2 (10)	2 (10)	2 (10)	3 (10)	4 (20)	5 (20)	—
Sarcopenic	1 (10)	1 (10)	1 (10)	1 (10)	2 (20)	2 (20)	—
Severe sarcopenic	1 (10)	1 (10)	0 (10)	1 (10)	1 (20)	2 (10)	—

^a^ Significant difference between scores of men and women.

β, stage of the sarcopenia is determined based on the advice of the European workgroup of sarcopenia; ALMI, appendicular muscle mass index; CON, control group; CT, combined training group; INI, integrase inhibitors; NNRTI, nonnucleoside reverse transcriptase inhibitors; NRTI, nucleoside reverse transcriptase inhibitors; PI, protease inhibitors.

### Primary outcome

#### Diagnostic measures of sarcopenia

#### Grip strength

Groups were similar at baseline, but following the intervention, grip strength increased for CT (∆ = 7.86 ± 8.50 kg, *P* = 0.001) and remained unchanged in the CON group (*Table* 3). A significant Group × Time interaction was found for grip strength (*P* = 0.001), indicating an increase in grip strength for CT compared with CON. Results of the ANOVA with stratification by sex revealed a Group × Time interaction for grip strength (*P* = 0.001), with the CT groups for women and men showing a greater change compared with their respective CON groups. The magnitude of the increase in grip strength in the CT groups was greater for men compared with women [10.40 ± 6.01 kg vs. 7.80 ± 5.26 kg; *P* = 0.03].

**Table 3:** Change scores (Δ) in body composition, ALMI, inflammatory markers, grip strength, and gait speed after 6 months of exercise training intervention in PLWH, adjusted for the preintervention values, LBM, fat mass, height, and BMI; mean ± SD [95% CI]

	CON‐men (*n* = 10) Δ (post‐pre)	CT‐men (*n* = 10) Δ (post‐pre)	CON‐women (*n* = 10) Δ (post‐pre)	CT‐women (*n* = 10) Δ (post‐pre)	CON (*n* = 20)	CT (*n* = 20)
Anthropometry						
BMI (m/kg^2^)	−1.08 ± 1.99 [−2.51 to 0.33]	−0.64 ± 3.36 [−3.04 to 1.76]	0.04 ± 0.14 [−0.09 to 0.17]	−0.29 ± 0.41 [−1.4 to 0.85]	−0.62 ± 1.60 [−1.44 to 0.20]	−0.52 ± 2.74 [−2.04 to 0.99]
Blood markers						
IL‐6 (pg/mm^3^)	1.17 ± 1.09^b^ [0.39 to 1.95]	−1.37 ± 0.51^b^ [−1.73 to −1.00]	1.26 ± 0.91^b^ [0.41 to 2.11]	−1.18 ± 0.49^b^ [−1.79 to −0.57]	1.21 ± 0.99^c^ ^,^ ^b^ [0.70 to 1.72]	−1.30 ± 0.49^c^ ^,^ ^b^ [−1.58 to −1.03]
TNF‐α (pg/mm^3^)	1.65 ± 1.58^b^ [0.52 to 2.79]	−1.17 ± 0.66^b^ [−1.64 to −0.69]	2.18 ± 1.13^b^ [1.13 to 3.24]	−1.90 ± 0.59^b^ [−2.64 to −1.16]	1.87 ± 1.40^c^ ^,^ ^b^ [1.15 to 2.59]	−1.41 ± 0.71^c^ ^,^ ^b^ [−1.81 to −1.01]
IGF‐1 (pg/mm^3^)	8.51 ± 87.57 [−54.13 to 71.16]	83.22 ± 64.25^b^ [37.26 ± 129.19]	5.32 ± 120.94 [−106 to 117.17]	56.62 ± 36.75^a^, ^b^ [10.98 to 102.26]	7.19±99^c^ [−43.70 to 58.10]	74.36 ± 56.64^c^ ^,^ ^b^ [−42.98 to 105.73]
Myostatin (pg/mm^3^)	−2.56 ± 107.70 [−79.61 to 74.48]	−136.39 ± 39.88^b^ [−164.92 to −107.86]	−101.56 ± 182.17 [−270.05 to 66.91]	−200.90 ± 78.03^b^ [−300.81 to −106.98]	−43.33 ± 146.60^c^ [−118.71 to 32.04]	−158.90 ± 62.03^c^ ^,^ ^b^ [−193.24 to −124.54]
Measures of sarcopenia						
Handgrip strength (kg)	−2.70 ± 3.38^b^ [−6.34 to −1.43]	10.40 ± 6.01^b^ [0.89 to 10.50]	−0.48 ± 3.29 [−3.58 to 2.53]	6.38 ± 5.26^a^, ^b^ [0.82 to 9.80]	−1.58 ± 2.47^c^ [−2.86 to −0.31]	7.86 ± 8.50^c^ ^,^ ^b^ [0.82 to 8.57]
Gait speed (m/s^2^)	−0.06 ± 0.04 [−0.10 to 0.006]	0.13 ± 0.08^b^ [0.06 to 0.19]	−0.04 ± 0.05 [−0.10 to 0.33]	0.20 ± 0.03^b^ [0.16 to 0.25]	−0.06 ± 0.52^c^ [−0.08 to −0.032]	0.16 ± 0.07^c^ ^,^ ^b^ [0.11 to 0.20]
ALMI (kg/m^2^)	−0.07 ± 0.09^b^ [−0.14 to −0.01]	0.30 ± 0.16^b^ [0.09 to 0.51]	−0.16 ± 0.21^b^ [−0.37 to –0.03]	0.29 ± 0.11^b^ [0.21 to 0.03]	−0.11 ± 0.14^c^ [−0.42 to 0.01]	0.29 ± 0.13^c^ ^,^ ^b^ [0.26 to 0.66]
Pre‐sarcopenic	2 (10)	0 (10)	2 (10)	1 (10)	4 (20)	1 (20)
Sarcopenic	1 (10)	0 (10)	1 (10)	0 (10)	2 (20)	0 (20)
Severe sarcopenic	1 (10)	1 (10)	0 (10)	1 (10)	1 (20)	2 (20)

a Significant difference between change scores of men and women.

b Significant difference compared with baseline values *P* < 0.05.

c Significant Group × time interaction.

CON, control group; CT, combined training group.

#### Appendicular lean mass index

Groups were similar at baseline; however, ALMI increased in the CT group (∆ = 0.29 ± 0.13 kg/m^2^; *P* = 0.001) while remaining unchanged in the CON group following the intervention. A significant Group × Time interaction was present for ALMI (*P* < 0.001), indicating an increase in ALMI for CT compared with CON. Further, stratification of data by sex revealed a significant increase in ALMI in both women and men in the CT groups compared with the respective CON groups (both *P* < 0.05). The magnitude of the increase in ALMI in the CT groups was higher among the men than women (0.30 ± 0.16 kg/m^2^ for men vs. 0.29 ± 0.11 kg/m^2^ for women; *P* = 0.042).

#### Gait speed

Groups were similar at baseline; however, gait speed increased in the CT group (∆ = 0.16 ± 0.07 m/s^2^; *P* < 0.001) and remained unchanged in the CON group following the intervention (*Table* 3). A significant Group × Time interaction was present for gait speed, showing an increase in gait speed for CT compared with CON following the intervention. There were no differences in the magnitude of the increase between sexes for gait speed in the CT group.

#### Prevalence of the sarcopenia

Based on the criteria from the EWGSOP 2(20), at baseline in the CT group, two men (20%) and three women (30%) were categorized as pre‐sarcopenic, one man (10%) and one woman (10%) were categorized at the sarcopenic stage, and one man (10%) and one women (10%) were categorized as severe sarcopenic. Following the intervention in the CT group, the presence of sarcopenia decreased to 0 in both men and women, representing a 10% improvement in both sexes. Pre‐sarcopenic incidence reduced to 0 and 1 in men and women, respectively, representing a 20% improvement in both sexes, and severe sarcopenia remained unchanged. The incidence of sarcopenia remained unchanged in participants of the control group over the course of the study.

### Secondary outcomes

#### Blood markers

#### Inflammatory biomarkers

At baseline, there were no significant differences in IL‐6 and TNF‐α between groups (*Table* 3). After the intervention IL‐6 and TNF‐α increased in the CT group (∆ = −1.30 ± 0.49 and ∆ = −1.41 ± 0.71, respectively, *P* < 0.001) and decreased in the CON group (∆ = 1.21 ± 0.99 and ∆ = 1.87 ± 1.40, *P* < 0.001). A significant Group × Time interaction was found for IL‐6 and TNF‐α (*P* < 0.001), indicating a reduction in these inflammatory biomarkers in the CT group compared with the CON group, with no sex differences.

#### IGF‐1

At baseline, there were no significant differences in IGF‐1 between groups (*Table* 3) but increased after the intervention in the CT group (∆ = 74.36 ± 56.64 pg/mm^3^, *P* < 0.001) and remained unchanged in the CON group. A significant Group × Time interaction was found for IGF‐1 (*P* < 0.001), showing an increase for the CT compared with the CON group. The magnitude of the increase was higher for the men compared with women (83.22 ± 64.25 vs. 56.62 ± 36.75 pg/mm^3^, *P* < 0.001).

#### Myostatin

At baseline, there were no significant differences in myostatin between groups (*Table* 3), but increased after the intervention in the CT group (∆ = −158.90 ± 62.03 pg/mm^3^, *P* < 0.001) and remained unchanged in the CON group. A significant Group × Time interaction was found for myostatin (*P* < 0.001), showing a reduction in CT compared with the CON group. The magnitude of reduction in myostatin was not different between men and women in the CT groups.

### Correlation analysis

**Figure 2:**
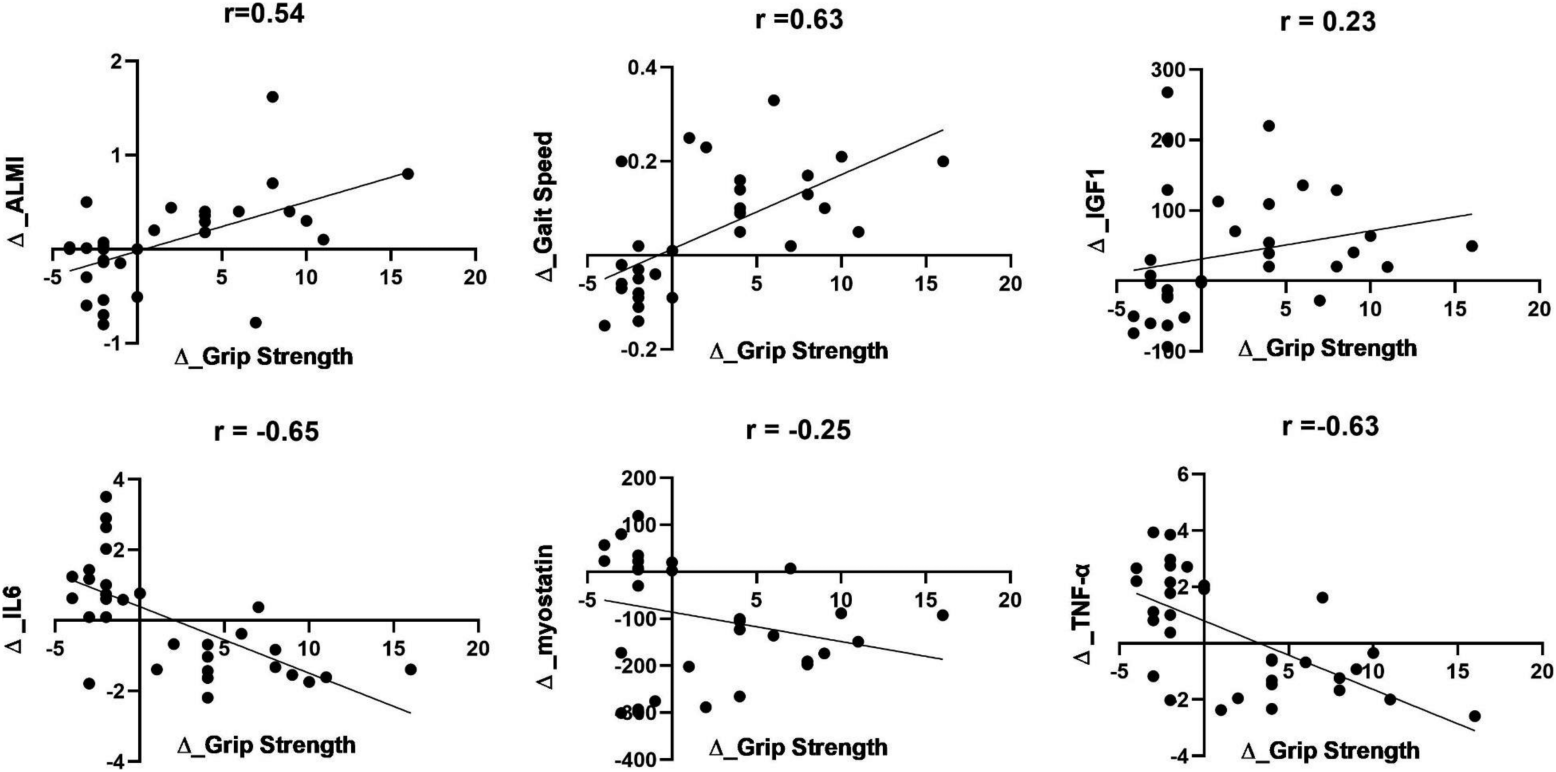
Correlation between change scores (Δ) of grip strength and gait speed, ALMI, growth factors and inflammatory markers.

A strong inverse correlation was found between grip strength and IL‐6 change scores (*r* = −0.65, *P* < 0.001) (*Figure* 2). The same pattern was observed between grip strength and TNF‐α change scores (*r* = −0.63, *P* < 0.001). A strong positive correlation was found between grip strength and ALMI change scores (*r* = 0.54, *P* = 0.001). Similarly, a strong positive correlation was found between grip strength and gate speed change scores (*r* = 0.63, *P* < 0.001). The grip strength change score was not related to the change scores for IGF‐1 and myostatin.

### Safety and compliance

Except for the usual muscle soreness and cramps that usually occur at the commencement of exercise training (especially resistance training), no adverse events were recorded throughout the study. Compliance with the exercise sessions was high for both men and women, with no differences between sexes in the attendance of training sessions. (85.2 ± 2.1% vs. 89.4 ± 2.3%, for men and women, respectively).

## Discussion

The primary aim of the present study was to examine the effectiveness of combined training on the diagnostic measures of sarcopenia, including grip strength, gait speed, and ALMI in PLWH. Examining changes in inflammatory markers and growth factors related to the development of sarcopenia was a secondary aim. Six months of combined training compared with the control condition resulted in greater grip strength, ALMI, and gait speed, as well as favourable changes in pro‐inflammatory cytokines. Consequently, the number of pre‐sarcopenic conditions in the combined training group was reduced but did not change in the control group. These findings are clinically relevant and show that combined training is an effective treatment for counteracting HIV‐associated sarcopenia, particularly in the early stages of sarcopenia.

### Grip strength

Grip strength is an indicator of the overall strength and is considered a good predictor of health in clinical populations.^19^ However, there is a paucity of research examining the effects of the exercise‐based intervention on grip strength in PLWH. It has been proposed that HIV infection and ART negatively impact muscular strength in PLWH. Loss of muscle strength in PLWH is suggested to be accompanied by adverse health outcomes and an increased risk of premature death.^20^ The present study finding of greater grip strength following combined training is consistent with the strength improvements reported in previous exercise studies in PLWH.^10^, ^21^ The improvement in muscular strength is likely to be attributed to neural adaptations and the accretion of skeletal muscle. This is partly supported by the strong positive correlation between change in grip strength and ALMI.

### Appendicular lean mass index

People living with HIV can live longer due to the advancement of ART; however, these medicines do not preserve muscle mass in PLWH and may exacerbate unfavourable body composition changes such as body fat redistribution.^3^, ^4^ The present study finding of increased ALMI following combined training is in agreement with reported increases in muscle mass following exercise interventions in PLWH.^7^, ^8^ This finding is clinically relevant because the skeletal muscle is a source of anti‐inflammatory cytokine and myokine secretion.^22^ Additionally, increased muscle mass in PLWH may improve their immune function^7^ and ability to perform activities of daily living (e.g. walking and navigating stairs).^23^

### Gait speed

Gait speed is an indicator of independence in daily life and lower limb strength and muscle mass in clinical populations. The present study found a significant improvement in gait speed following combined training with no difference between sexes. The effectiveness of combined training for enhancing gait speed is consistent with a previous study that reported these changes in older PLWH (50–75 years).^11^ Specifically, exercise focused on the development of muscle strength is important for improving the physical function of PLWH. This is supported by the strong positive correlation between changes in grip strength and gait speed found in the present study. Furthermore, an improvement in gait speed is clinically relevant because it is associated with better quality of life in PLWH, independent of other HIV‐related mortality risk factors.^20^

The rationale for selecting the TUG test to evaluate physical function changes following the intervention was partly based on this measure being among the recommended tests from the EWGSOP2.^15^ Additionally, the TUG test is widely used in the literature to predict sarcopenia in different clinical cohorts.^24^ However, this measure was developed to assess gait speed in older adults, has low specificity, and there is a ceiling effect on improvement.^25^ Therefore, the findings should be interpreted with caution, and future studies are needed to validate a context‐specific gait speed test in PLWH.

### Changes in pro‐inflammatory cytokines

Elevated levels of inflammatory cytokines are among the nominated contributors to the development of sarcopenia.^1^, ^4^ Specifically, elevated levels of TNF‐α and IL‐6 negatively affect muscle turnover, resulting in negative nitrogen balance and muscle loss. There is evidence that elevated levels of TNF‐α and IL‐6 can also directly impair the contractile function of the muscle, thus reducing muscle strength.^26^ Consequently, it has been suggested that a panel of circulatory inflammatory biomarkers should be used for the early detection of sarcopenia.^15^

In the present study, we observed a significant reduction in TNF‐α and IL‐6 following 6 months of combined training. Furthermore, the present study showed that increased grip strength was strongly associated with reductions in TNF‐α and IL‐6, hence corroborating the potential negative effect of these inflammatory cytokines on muscular strength. Our finding is in agreement with studies that previously have been shown the reduction of the inflammatory markers following exercise training in this cohort.^27^, ^28^ On the contrary, recently, a systematic review with meta‐analysis convicted by Zanetti *et al*. found that resistance training alone did not reduce IL‐6 and TNF‐α in PLWH.^9^ The discrepancy between these results and the present study findings may be due to combined training eliciting a more robust anti‐inflammatory response than resistance or aerobic training alone.^29^, ^30^ However, further studies are needed to determine the most effective training to improve the inflammatory profile of PLWH.

A detailed explanation of the mechanisms causing a reduction in inflammatory markers following physical training goes beyond the scope of the present study. However, the underpinning mechanism behind the anti‐inflammatory effect of the physical training is attributed to the secretion of the anti‐inflammatory myokines such as IL‐10 from working muscle. Additionally, reduction in adipose tissue, particularly abdominal adipose tissue, following physical training, will lead to a lower production of several pro‐inflammatory cytokines, such as TNF‐α.^31^

### Changes in growth factors

In the present study, we also measured the serum levels of IGF‐1 and myostatin as potential contributors to the development of sarcopenia. Over 6 months, there was a significant increase in the serum levels of the IGF‐1 following combined training, and the magnitude of the increase was higher among men than women. IGF‐1 is thought to mediate increases in muscle strength following resistance training in healthy adults.^32^ Although, the effect of IGF‐1 on the muscular strength of the PLWH appears to be more complex based on a lack of association between changes in grip strength and IGF‐1 in the present study. The exercise intervention was also able to reduce myostatin levels, which is a negative regulator of muscle growth. This result is a novel finding because no study to date has examined the myostatin response to an exercise‐based intervention in PLWH. However, the result is in agreement with studies that have examined the effects of exercise training in other clinical cohorts.^33^, ^34^

### Incidence of sarcopenia

A clinically relevant finding of the present study was the reduction in the number of participants diagnosed by one of the three stages of sarcopenia at baseline, following combined training. This promising finding suggests that combined training at the early stages of sarcopenia can halt or even reverse its progression in PLWH.

One unprecedented observation in the present study was the high prevalence of sarcopenia among the participants with HIV (40%, *n* = 16/40), which has been reported to be approximately 24% in this clinical cohort.^1^ One explanation could be the sociodemographic characteristics of participants from Iran in the present study, with the majority coming from lower‐income and lower education social classes. Malnutrition and unhealthy habits such as smoking and drinking are highly prevalent in Iranian PLWH and are suggested factors associated with the higher incidence of sarcopenia.^1^, ^35^ Another explanation for the high prevalence of sarcopenia of the study participants could be the relatively small sample size which might have inflated the actual sarcopenia incident. Therefore, future research with larger sample size is warranted to determine the exact prevalence of sarcopenia in Iranian PLWH.

### Adherence rate

One of the strengths of the current study was the high adherence rate which is contrary to most of the previous exercise‐based interventions in PLWH.^36^ The high adherence rate of the current study is attributable to the time of training sessions being flexible and the location of the sessions being close to where they would receive regular care. Additionally, participants that had difficulty travelling to the training site were provided with transportation.

### Strength and limitations

It must be acknowledged that the present study is not without limitations. First, similar to other studies in this cohort, the relatively small sample size prevents results from being generalized to the broader population of PLWH. Second, lifestyle factors such as diet were not controlled, which could influence the presence of sarcopenia and changes following the intervention. However, participants were asked to continue with their previous lifestyle and diet and avoid nutritional supplements during the study period. Third, we did not measure CD4 + (e.g. CD4 + nadir, current CD4+, CD4+/CD8 ratio+) among the participants, which is important to advance the understanding of immune function and development of sarcopenia in PLWH. Finally, the utilized diagnostic measure for sarcopenia in the present study is not designed to diagnose sarcopenia in PLWH. Therefore, it is possible that the tool used may overestimate or underestimate sarcopenia in PLWH. Future research is warranted to determine the context‐specific sarcopenia definitions in PLWH. The limitations addressed here do not diminish the study's novelty, with the results contributing to advance knowledge of the effectiveness of exercise on the diagnostic measures of sarcopenia in PLWH.

## Conclusion

In summary, the present study's findings suggest that combined training can be considered a feasible approach to enhance ALMI, reduce circulatory levels of pro‐inflammatory cytokines and improve muscle function in PLWH. Therefore, it is strongly recommended that health care providers consider physical training as a protective approach against sarcopenia in PLWH.

## Funding

The Tehran University of Medical Sciences supported this work, and Morteza Ghayomzadeh is supported by Murdoch Strategic Scholarship.

## Conflict of interest

The authors certify that they comply with the ethical guidelines for authorship and publishing of the *Journal of Cachexia, Sarcopenia, and Muscle*.^37^ Morteza Ghayomzadeh, Daniel Hackett, SeyedAhmad SeyedAlinaghi, Mohammad Gholami, Negin Hosseini Rouzbahani, and Fabrício Azevedo Voltarelli declare that they have no conflict of interest.
